# Determination of a radotinib dosage regimen based on dose–response relationships for the treatment of newly diagnosed patients with chronic myeloid leukemia

**DOI:** 10.1002/cam4.1436

**Published:** 2018-03-25

**Authors:** Hayeon Noh, Su Young Jung, Jae‐Yong Kwak, Sung‐Hyun Kim, Suk Joong Oh, Dae Young Zang, Suhyun Lee, Hye Lin Park, Dae Jin Jo, Jae Soo Shin, Young Rok Do, Dong‐Wook Kim, Jangik I. Lee

**Affiliations:** ^1^ Department of Pharmacy College of Pharmacy Yonsei University Incheon Korea; ^2^ Department of Pharmacy College of Pharmacy Seoul National University Seoul Korea; ^3^ Research Institute of Pharmaceutical Sciences Seoul National University Seoul Korea; ^4^ Chonbuk National University Medical School & Hospital Jeonju Korea; ^5^ Department of Internal Medicine Dong‐A University College of Medicine Busan Korea; ^6^ Department of Internal Medicine Kangbuk Samsung Hospital Sungkyunkwan University School of Medicine Seoul Korea; ^7^ Department of Internal Medicine Hallym University Sacred Heart Hospital Anyang Korea; ^8^ Central Research Institute IL‐YANG Pharmaceutical Co., Ltd. Yongin Korea; ^9^ Department of Medicine Dongsan Medical Center Keimyung University Daegu Korea; ^10^ Seoul St. Mary's Hospital Leukemia Research Institute The Catholic University of Korea Seoul Korea

**Keywords:** Chronic myeloid leukemia, dose determination, dose–response relationship, radotinib, tyrosine kinase inhibitor

## Abstract

Radotinib is a second‐generation *BCR‐ABL1* tyrosine kinase inhibitor approved for the treatment of chronic myeloid leukemia in chronic phase (CP‐CML). Here, using the data from a Phase 3 study conducted in patients with newly diagnosed CP‐CML, the dose–efficacy as well as dose–safety relationship analyses were performed to determine a safe and effective initial dosage regimen of radotinib. A significant positive association was detected between the starting dose of radotinib adjusted for body weight (Dose/BW) and the probability of dose‐limiting toxicity (≥grade 3 hematologic and nonhematologic toxicity) (*P *=* *0.003). In contrast, a significant inverse association was discovered between Dose/BW and the probability of major molecular response (*BCR‐ABL1/ABL1 *≤* *0.1%) when controlled for sex (*P *=* *0.033). Moreover, frequent dose interruptions and reductions secondary to radotinib toxicities occurred in the Phase 3 study, resulting in nearly half (44%) of patients receiving a reduced dose at a 12‐month follow‐up. In conclusion, the results of this study demonstrate the need for initial radotinib dose attenuation to improve the long‐term efficacy and safety of radotinib. Hence, the authors suggest a new upfront radotinib dose of 400 mg once daily be tested in patients with newly diagnosed CP‐CML.

## Introduction

The *BCR‐ABL1* tyrosine kinase inhibitors (TKIs) are the mainstay for the treatment of chronic myeloid leukemia in chronic phase (CP‐CML) 1, 2, 3. The *BCR‐ABL1* TKIs are typically administered at a fixed dose regimen irrespective of the patient's body size; however, it has been shown that the interpatient variability in drug exposure may contribute to the different responses to TKIs 4, 5, 6, 7. Studies have suggested the impact of the body weight (BW) or the body surface area (BSA) on the efficacy and/or safety outcomes of TKI treatments, particularly in Asian patients whose body size is relatively smaller than that of Caucasian patients. In general, a smaller body size appears to be associated with worse safety outcomes, whereas the influence of body size on the efficacy outcomes is somewhat less clear. For instance, the patients with smaller body size experienced significantly more toxicity from taking imatinib, sorafenib, and sunitinib than those with relatively larger body size 8, 9, 10, 11, 12. In two other large prospective trials conducted in Japan, a lower BW was identified as a risk factor for dose reductions and discontinuations associated with imatinib intolerance 13, 14. Moreover, the dose of imatinib divided by the patient's BW or BSA was correlated with the trough concentrations and the efficacy of imatinib 13, 15. The subanalyses of IRIS (the International Randomization Study of Interferon and STI571) and TOPS (the Tyrosine Kinase Inhibitor Optimization and Selectivity) trials have reported a correlation between patients’ BW or BSA and the trough concentrations of imatinib, which in turn was associated with the overall efficacy outcomes 4, 16. In consistence with those findings, our previous analysis on radotinib dose–safety response relationship using the data obtained from a Phase 2 study revealed that the starting dose of radotinib adjusted for individual patients’ BW (Dose/BW) at baseline was positively associated with the occurrence of dose‐limiting toxicity (DLT) by 12 months of radotinib therapy 17.

Radotinib (Supect^®^; IL‐YANG Pharmaceutical Co. Ltd., Yongin‐si, Gyeonggi‐do, Republic of Korea) is a selective second‐generation *BCR‐ABL1* TKI that was approved by the Korean Ministry of Food and Drug Safety in 2012 18. The approved starting dose is 400 mg twice daily for the treatment of patients with CP‐CML resistant and/or intolerant to imatinib, and 300 mg twice daily for the treatment of patients with newly diagnosed CP‐CML. Herein, using the clinical data collected from a Phase 3 study, the authors explored the relationships between the Dose/BW of radotinib and the efficacy (i.e., dose–efficacy response relationship) as well as the safety of radotinib (i.e., dose–safety response relationship). Based on the results of these analyses, we suggested an alternative initial dosage regimen of radotinib that would potentially reduce the incidence of adverse effects, allow uninterrupted radotinib treatment, and may enhance the long‐term effectiveness for the treatment of patients with CP‐CML. Given the prolonged period of TKI treatment for most CML patients during which low‐grade toxicities may be accumulated and become dose limiting, interfering with the chronic maintenance of treatment, the determination of a proper initial dose for radotinib is crucial. Compared with our previous study from which only the dose–safety response relationship was derived 17, this study provides more comprehensive evidence toward determining a proper radotinib dosage regimen by analyzing both the dose–efficacy and dose–safety response relationships using a larger patient population and incorporating the assessment of potential confounding factors.

## Methods

### Patients and study design

Dose–efficacy as well as dose–safety relationship analyses were performed using the clinical data collected for 12 months from a multinational, randomized, active‐controlled Phase 3 study conducted in 241 Asian patients with newly diagnosed CP‐CML (RERISE study) 19. The study protocol complied with the Declaration of Helsinki and was approved by the institutional review boards of all participating clinical trial centers. All patients enrolled in the study signed the informed consent before participating in the study. A detailed description of the Phase 3 study is reported elsewhere 19. Briefly, the patients were randomly assigned to receive a dose of radotinib 300 mg twice daily (total daily dose of 600 mg, *n* = 79), radotinib 400 mg twice daily (total daily dose of 800 mg, *n* = 81), or imatinib 400 mg daily (*n* = 81). The primary efficacy endpoint was the achievement of major molecular response (MMR) by 12 months, defined as the *BCR‐ABL1/ABL1* transcript ratio of 0.1% or less on the international scale based on a real‐time quantitative polymerase chain reaction using a peripheral blood specimen. The *BCR‐ABL1/ABL1* transcript was measured at regular intervals (at screening, and at 3, 6, 9, and 12 months). Dose‐limiting toxicity (DLT) was defined as a grade 3 or greater hematologic and nonhematologic toxicity according to the National Cancer Institute Common Terminology Criteria for Adverse Events (NCI‐CTCAE) version 3.0 20. Upon the occurrence of DLT, the administration of radotinib was interrupted until recovery to either grade 1 toxicity or less, after which the dose was reduced in a stepwise manner (i.e., 400 mg twice daily reduced to 300 mg twice daily, and 300 mg twice daily reduced to 200 mg twice daily). Radotinib therapy was permanently discontinued if the toxicity was not reduced to grade 1 or less within 28 days, or if more than two levels of dose reductions were required.

### Statistical analysis

In constructing the datasets for statistical analyses, each patient's Dose/BW was calculated using the total daily dose of radotinib administered at study initiation (600 mg or 800 mg) divided by the patient's BW at baseline. A logistic regression model was developed to explore the associations between the Dose/BW of radotinib and the achievement of MMR at 12 months (i.e., dose–efficacy response relationship), as well as the occurrence of DLT by 12 months (i.e., dose–safety response relationship) of radotinib therapy. In developing a multiple logistic regression model, potential confounding factors (e.g., age, sex, and baseline laboratory parameters such as hemoglobin, white blood cell counts, platelet counts, albumin, serum creatinine, total bilirubin, alanine transaminase, and aspartate transaminase) that were identified in univariate analyses were added to the model in a stepwise fashion on the basis of a statistically significance level of 0.05. Among the variables tested, sex was treated as a categorical variable (female = 0, male = 1), whereas all other variables were treated as continuous variables.

A receiver operator characteristic (ROC) analysis was conducted to assess the predictability of the Dose/BW for achieving MMR or developing DLT and to identify an optimal cutoff value of Dose/BW that would best distinguish patients with a low and a high likelihood of achieving MMR or developing DLT. The best distinguishing cutoff value was selected when Youden's index value (sensitivity + [specificity − 1]) was highest. Using this cutoff, patients were divided into a “low‐dose” and a “high‐dose” group after which the rates of MMR and DLT were compared using a chi‐squared test. In the dose–efficacy response relationship analysis, patients were excluded if radotinib was permanently discontinued for any reason within the 12 months of the study period or if their *BCR‐ABL1/ABL1* transcript data at 12 months were not available. In the dose–safety response relationship analysis, patients were excluded if they had dropped out of the study for any reason other than drug toxicity such as treatment failure, lost to follow‐up, or consent withdrawal.

A Kaplan–Meier method was employed with a log‐rank test to estimate and compare the time to first DLT between the low‐ and high‐dose groups. The time to first DLT was defined as the elapsed time from the date of first radotinib administration to the date of first DLT occurrence. The time to first DLT was censored for the patients who did not experience any DLT by 12 months or whose radotinib therapy was permanently discontinued for any reason unrelated to drug toxicity. The median time to first DLT was defined as the time (days) at which a half of patients experienced their first DLT. All statistical testing was performed using the SAS software version 9.4 (SAS Institute Inc., Cary, NC). The *P* values <0.05 were considered statistically significant.

## Results

A total of 160 patients who received radotinib therapy in the Phase 3 study were eligible for inclusion in the dose–response relationship analyses (Table 1). The patients consisted of 61 females and 99 males and had the mean ± standard deviation (SD) age of 47.3 ± 15.8 years (range, 18–84 years), baseline BW 62.9 ± 11.6 kg (range, 40–100 kg), and Dose/BW 11.5 ± 2.69 mg/kg/day (range, 6–20 mg/kg/day). Based on the international classification of body mass index (BMI) 21, 120 patients (75%) were in the normal range with respect to their BMI (18.5–24.9 kg/m^2^), 30 patients (19%) overweight (25–29.9 kg/m^2^), five patients (0.03%) obese (≥30 kg/m^2^), and five patients (0.03%) underweight (<18.5 kg/m^2^).

**Table 1:** Baseline characteristics of the radotinib‐treated patients (*n* = 160) in Phase 3 study

Sex, *n* (%)	
Male	99 (61.9)
Female	61 (38.1)
Age (years)	
Mean ± SD	47 ± 16
Median (range)	45 (18–84)
Race, *n* (%)	
Korean	141 (88.1)
Thai	8 (5.0)
Filipino	6 (3.8)
Indo‐Malayan	4 (2.5)
Chinese	1 (0.6)
Sokal score, *n* (%)	
Low (relative risk < 0.8)	43 (26.9)
Intermediate (0.8 ≤ relative risk ≤ 1.2)	76 (47.5)
High (relative risk > 1.2)	41 (25.6)
Body weight, kg	
Mean ± SD	62.9 ± 11.6
Median (range)	60.7 (40–100)
Dose/BW, mg/kg/day	
Mean ± SD	11.5 ± 2.69
Median (range)	11.1 (6–20)
BMI, kg/m^2^	
Mean ± SD	23.0 ± 3.1
Median (range)	22.8 (16.4–32.1)

BMI, body mass index; Dose/BW, total daily dose of radotinib adjusted for body weight; SD, standard deviation.

### Dose–efficacy response relationship

Among the eligible 160 patients, 126 patients were included in the dose–efficacy response relationship analysis. Excluded patients either had permanently stopped radotinib therapy before completing the follow‐up by 12 months (*n* = 33) or did not have the *BCR‐ABL1/ABL1* transcript data at 12 months (*n* = 1). Of the 126 patients, approximately a half of the patients (*n* = 64) achieved MMR at 12 months.

A logistic regression model developed demonstrated a trend for an inverse relationship between Dose/BW and the probability of achieving MMR at 12 months. Among the potential confounding factors tested, sex was associated with MMR in a univariate analysis (*P *=* *0.046). When a multiple logistic regression analysis was applied, the relationship could be expressed as the following equation:

$$\begin{array}{cc} \text{Logit}(P)=\ln[P/(1-P)]\\ & =-0.21\times (\text{Dose}/\text{BW})-0.88\times (\text{Sex})+2.90 \end{array}$$

**Figure 1:**
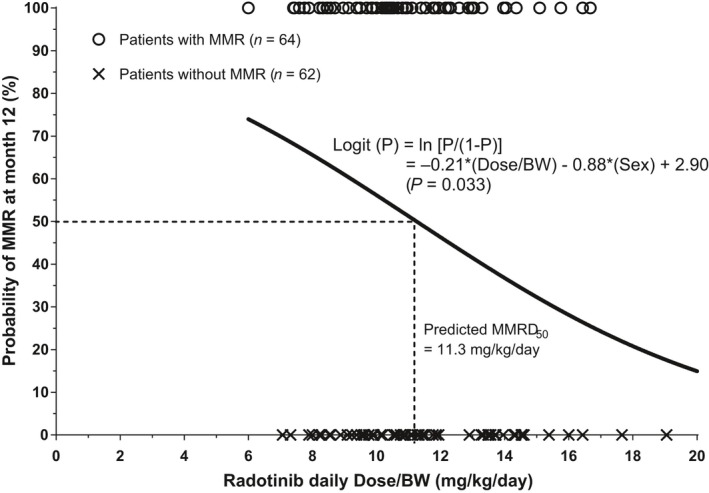
Logistic regression curve that predicts the probability of major molecular response (MMR) at 12 months in relation to the daily dose of radotinib adjusted for patients’ baseline body weight (Dose/BW), when controlled for sex. *P* indicates the probability that a patient achieves MMR.

The *P* represents the probability that a patient achieves MMR. The *P* values for the regression model, coefficients for the Dose/BW, sex, and intercept are 0.033, 0.019, 0.048, and 0.013, respectively (Fig. 1). The adjusted odds ratio (OR) for the Dose/BW was 0.81 (95% confidence interval [CI], 0.68–0.97), meaning that a unit increase in the Dose/BW was associated with a 19% decrease in the odds of achieving MMR after controlling for sex. The estimated Dose/BW for the 50% probability of MMR (MMRD_50_) was 11.3 mg/kg/day. Other variables including age (*P *=* *0.507), hemoglobin (*P *=* *0.079), white blood cell counts (*P *=* *0.170), platelet counts (*P *=* *0.067), albumin (*P *=* *0.253), serum creatinine (*P *=* *0.769), total bilirubin (*P *=* *0.896), alanine transaminase (*P *=* *0.932), and aspartate transaminase (*P *=* *0.951) were not significantly associated with the achievement of MMR at 12 months and therefore were not included in the model. A subsequent ROC analysis resulted in the area under the curve (AUC) of 0.64 (95% CI, 0.54–0.74), and the optimal cutoff dose of 13.3 mg/kg/day based on Youden's index to predict MMR achievement at 12 months. When the patients were divided into a “low‐dose” and a “high‐dose” group using the cutoff value of 13 mg/kg/day, a significantly higher proportion of patients achieved MMR at 12 months in the low‐dose group than in the high‐dose group (55.7% vs. 34.5%, *P *=* *0.045, chi‐squared test).

### Dose–safety response relationship

**Figure 2:**
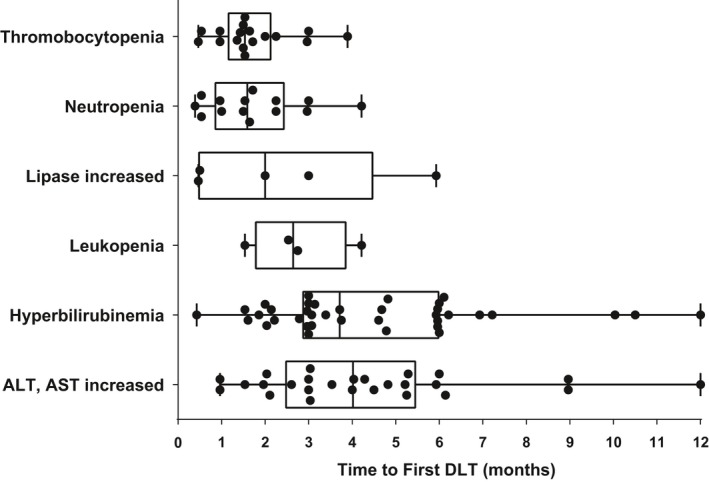
Box plots showing distributions of time to first dose‐limiting toxicity (DLT) categorized by DLT manifestations. The vertical line across the box is the median, and the box represents the interquartile range (IQR; 25th to 75th percentile). The bars extend to the minimum and the maximum values. Black dots indicate the individual data points.

Five patients were excluded from the dose–safety response relationship analysis who had dropped out of the study for any reason other than drug toxicity such as treatment failure, lost to follow‐up, and consent withdrawal. Of the remaining 155 patients, 103 patients (67.5%) experienced at least one episode of DLT by 12 months. The types of first‐occurring DLT include, in the order of frequency, hyperbilirubinemia (37 episodes), elevations in liver enzymes (26 episodes), thrombocytopenia (17 episodes), neutropenia (14 episodes), elevations in lipase (5 episodes), leukopenia (4 episodes), itching (3 episodes), fatigue (2 episodes), nausea (2 episodes), and others. Eighteen patients experienced two or more different types of DLTs simultaneously at the time of first DLT occurrence, among which nine patients had a combination of hematologic toxicities only, eight had a combination of nonhematologic toxicities only, and one had both hematologic and nonhematologic toxicities. In general, hematologic toxicities occurred relatively earlier than nonhematologic toxicities (Fig. 2). Whereas almost all episodes of thrombocytopenia (16 of 17), neutropenia (13 of 14), and leukopenia (3 of 4) appeared during the first 3 months of radotinib therapy, only approximately a third of episodes of hyperbilirubinemia (14 of 37) and liver enzyme elevation (9 of 26) occurred during the same time frame. The median time to first DLT was 43 (interquartile range, 32.5–59.5) days for thrombocytopenia, 44.5 (24–68) days for neutropenia, 56 (13.5–125) days for lipase elevation, 74 (50–107.8) days for leukopenia, 104 (80.5–167.5) days for hyperbilirubinemia, and 112.5 (69.5–152.5) days for liver enzyme elevation.

Using a logistic regression analysis, a significantly positive association was discovered between the Dose/BW of radotinib and the probability of first DLT by 12 months. The probability could be estimated with the following equation:

Logit(P)=ln[P/(1−P)]=0.21×(Dose/BW)−1.62

**Figure 3:**
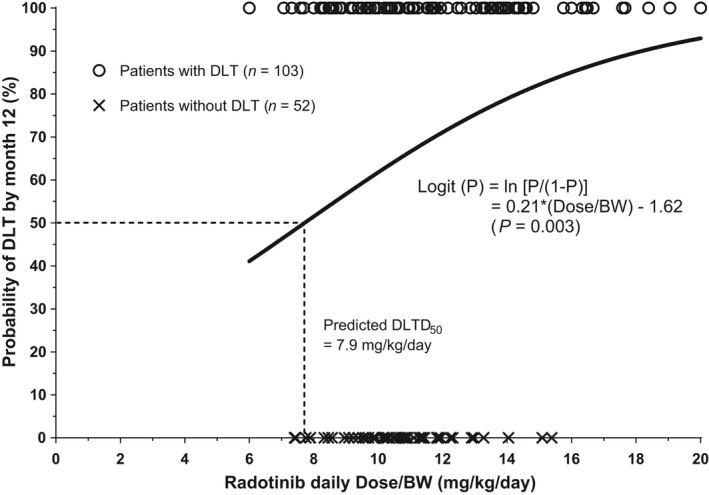
Logistic regression curve that predicts the probability of first dose‐limiting toxicity (DLT) by 12 months in relation to the daily dose of radotinib adjusted for patients’ baseline body weight (Dose/BW). *P* indicates the probability that a patient experiences DLT.

The *P* represents the probability that a patient experiences DLT. The *P* values for the regression model, coefficients for the Dose/BW, and intercept are 0.003, 0.005, and 0.049, respectively (Fig. 3). The OR for the Dose/BW was 1.23 (95% CI, 1.06–1.42), indicating that a unit increase in Dose/BW was associated with a 23% increase in the odds of developing DLT. The estimated Dose/BW for the 50% probability of DLT (DLTD_50_) was 7.9 mg/kg/day. Other baseline patient variables including age (*P *=* *0.606), sex (*P *=* *0.082), hemoglobin (*P *=* *0.077), white blood cell counts (*P *=* *0.309), platelet counts (*P *=* *0.093), albumin (*P *=* *0.153), serum creatinine (*P *=* *0.248), total bilirubin (*P *=* *0.304), alanine transaminase (*P *=* *0.393), and aspartate transaminase (*P *=* *0.397) were not significantly associated with the occurrence of DLT and therefore were not included in the model. In the ROC analysis, an AUC of 0.62 (95% CI, 0.54–0.71) was observed with the distinguishing cutoff dose of 13.0 mg/kg/day based on Youden's index. When the patients were divided into a “low‐dose” and a “high‐dose” group using this cutoff dose, significantly more patients experienced DLT in the high‐dose group than in the low‐dose group (90.7% vs. 57.1%; *P *<* *0.0001, chi‐squared test).

**Figure 4:**
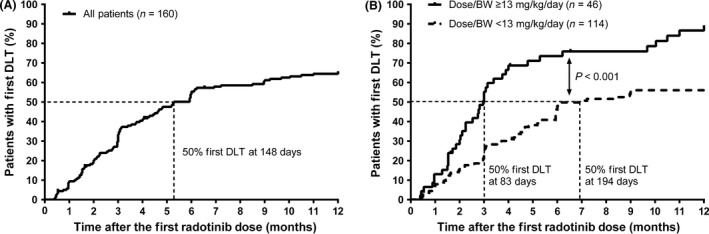
Kaplan–Meier curves for the time to first dose‐limiting toxicity (DLT) by 12 months (A) in all patients (*n* = 160) treated with radotinib in Phase 3 study, and (B) in the patients who received a daily dose of radotinib adjusted for patients’ baseline body weight (Dose/BW) ≥13 mg/kg/day compared with those who received a Dose/BW <13 mg/kg/day.

In a Kaplan–Meier analysis using all radotinib‐treated patients in the Phase 3 study (*n* = 160), the proportion of patients who experienced DLT increased steeply during the first 6 months then more modestly in the remaining study period (Fig. 4A). By 12 months, 66% of patients experienced at least one episode of DLT with the median time to first DLT of 148 days. The patients who received a Dose/BW of 13 mg/kg/day or higher had a significantly earlier onset of DLT than those who received lower than 13 mg/kg/day (median time to first DLT, 83 days and 194 days, respectively; *P *<* *0.001, log‐rank test) (Fig. 4B).

It is of note that a high rate of DLT occurred during the course of the Phase 3 study, especially in the first 3 months of radotinib initiation. Approximately two‐thirds (64%; *n* = 103) of patients in the radotinib arms experienced an interruption of treatment followed by a reduction in radotinib dose, or permanent treatment discontinuation owing to DLT. At the time of 12‐month follow‐up, of 79 patients in the arm of radotinib 600 mg/day, nearly a half of patients (44%; *n* = 35) had their dose reduced to 400 mg/day, 33 patients (42%) had their dose unchanged, and 11 patients (14%) had discontinued radotinib treatment. Of 81 patients in the arm of 800 mg/day, 23 patients (28%) and 12 patients (15%) were receiving a reduced dose of 600 mg/day and 400 mg/day, respectively, and 24 patients (30%) had discontinued treatment. The median dose of radotinib at 12‐month follow‐up was 400 mg/day and 600 mg/day for the arms of 600 mg/day and 800 mg/day, respectively.

## Discussion

The results of the present study suggest the need for lowering the starting dose of radotinib in patients with newly diagnosed CP‐CML. In terms of efficacy results, the probability of achieving MMR at 12 months was associated with the Dose/BW of radotinib in an inverse manner. That is, patients who received a lower Dose/BW at the initiation of radotinib treatment were likely to experience a better therapeutic response at 12 months than those who received a higher Dose/BW. Regarding safety, the results were similar to those of our previous study, in which a higher Dose/BW was associated with a significantly greater risk of DLT 17.

It appears paradoxical that the Dose/BW of radotinib and the probability of achieving MMR is inversely related. However, this seemingly inverse relationship may be attributable to the fact that the patients who received a higher Dose/BW initially were more likely to encounter DLT, necessitating dose interruptions and reductions that led to a lower efficacy response rate in the long run. Similarly, a lower body weight (<60 kg) was associated with a higher drug discontinuation rate and thus a lower efficacy response for imatinib as shown in another study 14. Other possible explanation for the inverse relationship is that a high radotinib dose may achieve the target protein binding faster but unnecessarily induce more “off‐target” effects, interfering with its efficacy 22. Other targeted agents such as imatinib and gefitinib have shown a similar dose–response pattern, in which doses above a certain level did not lead to a better tumor response, but rather toxicity increased in a dose‐dependent manner 23, 24, 25. In a study evaluating 24 Phase 1 studies for various targeted anticancer agents 26, major efficacy outcomes were similar across the low‐dose (≤25% of maximum tolerated dose, MTD), medium‐dose (25–75% of MTD) and high‐dose (≥75% of MTD) groups. However, the high‐dose group had a significantly higher rate of dropout secondary to toxicities compared with the low‐dose group. In the Phase 3 study of radotinib, frequent DLTs requiring dose interruptions and reductions were noted 19. This resulted in taking a reduced dose at the 12‐month follow‐up in nearly a half of patients in each radotinib arm (44% and 43% in the arms of 600 mg/day and 800 mg/day, respectively). Most of the newly occurring DLTs, particularly ≥grade 3 hematologic toxicities, occurred in the early treatment period (i.e., first 3 months), indicating that an upfront dose attenuation is needed.

In the present study, sex appears to be a confounding factor in the relationship between the Dose/BW of radotinib and MMR. This seems to be secondary to the Dose/BW difference between the female and male patients (13.1 mg/kg vs. 10.5 mg/kg; *P *<* *0.0001, Student's *t*‐test), and the influence of sex on the efficacy response. Sex was also marginally (*P *=* *0.082) associated with DLT in this study with a higher prevalence in females (77%) than in males (60%). Sex‐based differences in response to CML treatments have been reported with females generally showing more favorable treatment outcomes but a higher susceptibility to the adverse effects of TKIs 27, 28. This may be secondary to the higher trough concentrations and bioavailability of TKIs observed in female patients 4, 29, 30, which may be explained by the differences in the gastrointestinal transit and absorption 31, 32. Other variables were not significantly associated with MMR or DLT. This may be associated with the fact that the Phase 3 study protocol only allowed the inclusion of patients whose laboratory parameters were within prespecified limits. Perhaps less stringent inclusion criteria and a larger sample size would have detected the significant effects of such variables.

In our previous study 17, the authors had reported a dose–safety response relationship of radotinib using the clinical data obtained from a Phase 2 study and proposed a two‐tier weight‐based dosing approach to improve the safety profile of radotinib. Compared with the previous study, the present study using the data from a Phase 3 study included a larger number of patients and a different patient population (newly diagnosed vs. imatinib‐resistant CP‐CML). Moreover, the present study explored, in addition to the dose–safety response relationship, the dose–efficacy response relationship and included an assessment of potential confounding factors for the purpose of suggesting a more effective and safe dosage regimen for patients with newly diagnosed CP‐CML. By any means, the cutoff value of Dose/BW that best distinguishes the MMR achievement as well as the DLT occurrence in the Phase 3 study was 13 mg/kg/day, similar to the value of 12 mg/kg/day that distinguished the DLT occurrence in the Phase 2 study 17.

The limitations of this study are the retrospective data analyses, the lack of drug concentration data, and a relatively small sample size that may have weakened the strengths of the results. However, taken all evidence together, initiating radotinib at a lower dose appears desirable to alleviate toxicities and eventually to enhance the efficacy response to radotinib therapy. The issue of how much lower dose that can be administered needs further investigation. Based solely on the observed data, a dose as low as 8 mg/kg/day is anticipated to significantly reduce toxicities without compromising, and even enhancing, the efficacy response.

In conclusion, the results of the present study highlight that lowering the initial dose of radotinib in the treatment of patients with newly diagnosed CP‐CML will likely improve the efficacy and safety outcomes of radotinib treatment. Given the currently approved radotinib dose being 300 mg twice daily, the authors suggest 400 mg once daily be tested to patients as the first‐line treatment of CP‐CML. Such regimen is also likely to improve patients’ adherence to radotinib therapy. The dose may be titrated up later as needed based on a careful monitoring of efficacy and safety responses. Future studies are warranted to evaluate the clinical pharmacokinetics and pharmacodynamics of radotinib in patients and the tyrosine kinase inhibition activity of radotinib in leukemic cells for the suggested dosage regimen compared with other regimens. Such studies may clearly elucidate the radotinib dose–concentration–clinical response relationships and provide more rigorous evidence toward determining a proper dosage regimen for the treatment of CP‐CML.

## Conflicts of Interest

HN, SYJ, and JIL received research funding from IL‐YANG Pharm. Co., Ltd. for the conduct of the present work. HLP, DJJ, and JSS are current employees of IL‐YANG Pharm. Co., Ltd.
